# Elevated Levels of miR-144-3p Induce Cholinergic Degeneration by Impairing the Maturation of NGF in Alzheimer’s Disease

**DOI:** 10.3389/fcell.2021.667412

**Published:** 2021-04-09

**Authors:** Lan-Ting Zhou, Juan Zhang, Lu Tan, He-Zhou Huang, Yang Zhou, Zhi-Qiang Liu, Youming Lu, Ling-Qiang Zhu, Chengye Yao, Dan Liu

**Affiliations:** ^1^Department of Pathophysiology, School of Basic Medicine, Tongji Medical College, Huazhong University of Science and Technology, Wuhan, China; ^2^Collaborative Innovation Center for Brain Science, The Institute of Brain Research, Huazhong University of Science and Technology, Wuhan, China; ^3^Department of Neurology, Union Hospital, Tongji Medical College, Huazhong University of Science and Technology, Wuhan, China

**Keywords:** Alzheimer’s disease, NGF, miR-144-3p, cholinergic degeneration, synaptic disorder

## Abstract

Cholinergic degeneration is one of the key pathological hallmarks of Alzheimer’s disease (AD), a condition that is characterized by synaptic disorders and memory impairments. Nerve growth factor (NGF) is secreted in brain regions that receive projections from the basal forebrain cholinergic neurons. The trophic effects of NGF rely on the appropriate maturation of NGF from its precursor, proNGF. The ratio of proNGF/NGF is known to be increased in patients with AD; however, the mechanisms that underlie this observation have yet to be elucidated. Here, we demonstrated that levels of miR-144-3p are increased in the hippocampi and the medial prefrontal cortex of an APP/PS1 mouse model of AD. These mice also exhibited cholinergic degeneration (including the loss of cholinergic fibers, the repression of choline acetyltransferase (ChAT) activity, the reduction of cholinergic neurons, and an increased number of dystrophic neurites) and synaptic/memory deficits. The elevated expression of miR-144-3p specifically targets the mRNA of tissue plasminogen activator (tPA) and reduces the expression of tPA, thus resulting in the abnormal maturation of NGF. The administration of miR-144-3p fully replicated the cholinergic degeneration and synaptic/memory deficits observed in the APP/PS1 mice. The injection of an antagomir of miR-144-3p into the hippocampi partially rescued cholinergic degeneration and synaptic/memory impairments by restoring the levels of tPA protein and by correcting the ratio of proNGF/NGF. Collectively, our research revealed potential mechanisms for the disturbance of NGF maturation and cholinergic degeneration in AD and identified a potential therapeutic target for AD.

## Introduction

Alzheimer’s disease (AD) is a progressive neurodegenerative disease and the most common cause of dementia (Alzheimer’s Disease Facts and Figures, 2020). The main clinical symptoms of AD are progressive learning and memory deficits. Although researchers have revealed that the amyloid cascade and tauopathy play important roles in the pathogenesis of AD (Sotthibundhu et al., 2008; Knowles et al., 2009; Ovsepian et al., 2014), related drug discovery strategies and clinical trials have yet to meet with success. Until now, only five drugs have been approved by the Food and Drug Administration (FDA) for the therapy of AD; three of these (donepezil, galantamine, and rivastigmine) are cholinesterase inhibitors, thus suggesting that cholinergic dysfunction plays a critical role in the progression of AD. Previous researchers also reported that in the early stages of AD, it is the cholinergic synapses that are particularly affected by an overload of Aβ, rather than other types of synapses (Wong et al., 1999; Bell et al., 2006). In line with this, it was demonstrated that cholinergic neurons in the basal nucleus of Meynert were selectively affected in patients with AD (Whitehouse et al., 1981). Moreover, the transcription of choline acetyltransferase (ChAT) was severely repressed in existing cholinergic neurons (Wilcock et al., 1982). In the brain of aged AD transgenic mice (Tg2576), researchers observed strong staining of acetylcholinesterase (AChE) associated with dystrophic fibers within cholinergic projections (Apelt et al., 2002). Collectively, all of these abnormalities in the cholinergic systems show strong correlations with impaired synaptic/memory in AD (Oda, 1999). Therefore, it is important to investigate the precise mechanisms that cause such cholinergic dysfunction.

Nerve growth factor (NGF) is a key neurotrophic factor that is involved in the regulation of growth, maintenance, proliferation, and survival of cholinergic neurons (Niewiadomska et al., 2011). Specifically, NGF has been reported to elevate the activity and promote the expression of ChAT (Pongrac and Rylett, 1998), thus increasing the synthesis and release of acetylcholine and the expression of vesicular acetylcholine transporter (Oosawa et al., 1999). Previous work has shown that endogenous NGF is mainly generated in the hippocampi, the neocortex, and other targets within the basal forebrain (Johnston et al., 1987; Koliatsos et al., 1990; Zhang et al., 2013). Secreted NGF can be trafficked in a retrograde manner to basal forebrain cholinergic neurons (BFCNs) and plays an important role in modulating cholinergic synaptic transmission and hippocampal plasticity (Conner et al., 2009). In the AD brain, the levels of proNGF, the precursor of NGF, are known to be increased (Fahnestock et al., 1996), thus leading to a change in the proNGF/NGF ratio. Previous researchers suggested that proNGF is neurotoxic because it can bind with the p75 receptor to activate apoptotic pathways (Ioannou and Fahnestock, 2017). Numerous lines of evidence have suggested that the increased ratio of proNGF/NGF in the AD brain is due to the defective processing of proNGF into the mature form of NGF (Cuello and Bruno, 2007; Bruno et al., 2009a). This is because reduced levels of tissue plasminogen activator (tPA) and plasmin, the two key enzymes required for the processing of proNGF, have been detected in the brains of patients with mild cognitive impairments (Bruno et al., 2009a). These patients also had increased levels of MMP, an enzyme that degrades NGF (Bruno et al., 2009b). If we are to develop an NGF-based therapeutic strategy for AD, it is important that we strive to understand the mechanisms that are responsible for the deregulation of proNGF/NGF processing, particularly with regard to the reduction of tPA or plasmin.

In this study, we demonstrate that the elevation of miR-144-3p in the hippocampi and prefrontal cortex can directly inhibit the translation of its target, tPA. The loss of tPA results in an increase in the proNGF/NGF ratio, which then promotes cholinergic degeneration in a mouse model of AD. The artificial upregulation of miR-144-3p in wild-type mice fully simulated these cholinergic degeneration and synaptic/memory impairments. Finally, administration of the antagomir of miR-144-3p partially rescued the cholinergic degeneration and synaptic/memory impairments in AD mice by rebalancing the proNGF/NGF ratio.

## Materials and Methods

### Animals

APPswe/PS1dE9 mice (APP/PS1 mice) were purchased from the Jackson Laboratory (Bar Harbor, ME, United States, stock #034829) and conserved in the Experimental Animal Central of Tongji Medical College at Huazhong University of Science and Technology. The genotyping protocol was performed according to the manufacturer’s instructions, and wild-type littermates were used as control. The animals were bred at room temperature with food and water *ad libitum* on a 12-h light/dark cycle. All animal experiments were approved by the Animal Care and Use Committee of Tongji Medical College and under its guidelines.

### Cell Culture

The mouse neuroblastoma N2a cell line was maintained in DMEM supplemented with 10% fetal bovine serum (FBS) at 37°C in a 5% carbon dioxide (CO_2_) condition. The culture medium was replaced every 3 days. Cell transfection was performed by Lipofectamine 2000 (Invitrogen, Carlsbad, CA, United States) according to the manufacturer’s instruction.

### Context–Place Memory Test

#### Apparatus

The experimental device is two wooden boxes (30 × 50 × 50 cm), and their bottom and walls are, respectively, pasted paper with different patterns and colors to distinguish the two different contexts (1 and 2).

#### Procedure

Mice were familiarized to the experimental room for 30 min before the behavioral experiments and then were placed in context 1 for free exploration for 15 min. On the second day, the mice were habituated in context 2 for 15 min; the experimental process was the same as the first day. On the third day, the mice were habituated to explore contexts 1 and 2 for 5 min with a 1-h interval. Context 1 (2) was cleaned with 70% ethanol between each trial. On the fifth day, two objects with similar shape and color (A/B) were placed in contexts 1 and 2. Two of object A are placed on the left and north of context 1, and two of object B are placed on the right and north of context 2. Then the mice were placed in the center of contexts 1 and 2 for 2 min to explore the contexts and objects to learn the spatial arrangement of the objects that are associated with each context. On the test stage, two of object C are placed on the left and right of context 1 or 2, and the mice were allowed to explore context 1 or 2 for 2 min. Time spent exploring the novel object within familiar location (TF) and novel object within novel location (TN) was measured. The discrimination index was defined as follows: (TN − TF)/(TN + TF) × 100%. This behavioral paradigm was performed as previously reported (Lesburguères et al., 2017).

### Quantitative RT-PCR

The total RNA was extracted by a TRIzol reagent (Invitrogen, CA, United States) following the manufacturer’s instructions. One microgram of RNA was reversely transcripted for mRNA or miRNA by a First-Strand cDNA Synthesis Kit (TOYOBO, Osaka, Japan) or a miRcute Plus miRNA First-Strand cDNA Kit (Tiangen, Beijing, China), respectively. The standard qPCR was performed on an ABI StepOnePlus real-time quantitative PCR instrument using TB Green^®^ Premix Ex Taq^TM^ II (Takara, Tokyo, Japan). The reaction was performed with pre-denaturation at 95°C for 3 min, followed by 40 cycles of denaturation at 95°C for 5 s and annealing at 60°C for 30 s. This cycle was followed by a melting curve analysis, ranging from 60 to 95°C with temperature increases by steps of 0.5°C every 10 s. The primers used for RT-PCR detection were listed in Table 1.

**TABLE 1:** Primers.

**Gene**	**ID**	**qPCR Primer (from 5′ to 3′)**
mmu-miR-144-3p	MIMAT0000156	Forward Sequence: TACAGTATAGATGATGTACT Reverse Sequence: GCTGTCAACGATACGCTACG
U6	19862	Forward Sequence: GATGACACGCAAATTCGTGAA Reverse Sequence: GCTGTCAACGATACGCTACG
tPA	18791	Forward Sequence: GTTACACAGCGTGGAGGACCAA Reverse Sequence: CACGTCAGCTTTCGGTCCTTCA
NGF	18049	Forward Sequence: GTTTTGCCAAGGACGCAGCTTTC Reverse Sequence: GTTCTGCCTGTACGCCGATCAA
Plg	18815	Forward Sequence: CCTCATAGGCACAACAGGACAC Reverse Sequence: TGGCTGTCAGTGGTATAGCACC
β-Actin	11461	Forward Sequence: GAGACCTTCAACACCCCAGC Reverse Sequence: GGAGAGCATAGCCCTCGTAGAT

### Western Blot

Mice were sacrificed, and their brains were immediately dissected. The tissues were extracted with RIPA Lysis Buffer (Beyotime, Shanghai, China) with a protease inhibitor cocktail (Roche) on ice. After boiling for 10 min, the protein samples were lysed by 20 pulses of sonication, and the concentration of proteins was measured by a BCA Protein Assay Reagent (Thermo Fisher Scientific, IL, United States). Proteins were separated by 10% SDS-PAGE gel and transferred to nitrocellulose membranes (GE Healthcare Life Sciences, Loughborough, United Kingdom). After blocking in 5% non-fat milk for 30 min, the membranes were incubated with primary antibodies (Table 2) overnight at 4°C, followed by washing with phosphate-buffered saline with Tween 20 (PBST). Then the membranes were incubated with anti-rabbit or anti-mouse IgG-conjugated secondary antibodies IRDye 800 (1:10,000; Rockland Immunochemicals) for 1 h at room temperature. The protein bands were visualized by using the Odyssey Imaging System (LI-COR, Lincoln, NE, United States).

**TABLE 2:** Antibodies.

**Name**	**Source**	**Cat#**	**WB/IF/IHC**	**RRID**
ChAT	Millipore	AB144P	1:100 for IHC	RRID:AB_2079751
NGF	Abcam	ab52918	1:500 for WB	RRID:AB_881254
tPA	ABclonal	A4210	1:200 for IF, 1:1,000 for WB	RRID:AB_2863209
Plg	ProteinTech	66399-1-Ig	1:1,000 for WB	RRID:AB_2881773
β-Actin	ProteinTech	60008-1-Ig	1:3,000 for WB	RRID:AB_2289225

### Immunohistochemistry

The immunohistochemistry was performed according to the previously described protocol (Yan et al., 2018). Mice were anesthetized using a mixture of ketamine (100 mg/kg) and dexmedetomidine (0.5 mg/kg) with intraperitoneal injection. Then the mice were perfused with 0.9% normal saline followed by precooled 4% paraformaldehyde solution. The brains were carefully taken out of the cranial cavity and soaked in 4% paraformaldehyde solution overnight. After gradient dehydration of 30% sucrose solution, coronal slices were cut with a thickness of 30 μm in a freezing cryostat (SLEE, Mainz, Germany). Brain slices were rinsed with 1 × PBS for 10 min and incubated with 0.3% H_2_O_2_ and 0.5% Triton X-100 at room temperature for 30 min to break the membrane. After those section were rinsed with PBS solution thrice, the non-specific antigen was blocked by incubation with 5% bovine serum albumin (BSA) in 1 × PBS for 30 min at room temperature. The sections were then incubated with primary antibody goat-anti-ChAT (Merck Millipore, AB144P, 1:400) at 4°C overnight. The slices were rinsed with 1 × PBS thrice and incubated with the secondary antibody biotinylated anti-goat (Vector Laboratories, BA-9500, 1:300) diluted with PBS for 2 h at 37°C. They were washed in PBS thrice and added with streptomycin-labeled peroxidase working solution (ABC-kit, Vector Laboratories, PK-4500, 1:300) for 1 h at 37°C. After being washed in PBS, the brain slices were stained in 3,3′-diaminobenzidine (DAB) staining solution (D-8001, Sigma-Aldrich, Germany) for 5–10 min, and then PBS was added to stop the reaction. Then the brain slices were stuck on glass slides coated with gelatin. After being dried, they were dehydrated in a series of ethanol, 75, 80, 95, and 100% ethanol, for 10 min each. And they were made transparent in xylene and sealed. Digital images for all slices were taken with a Coolpix 5000 Nikon Camera.

### Fluorescence *in situ* Hybridization (FISH)

FISH was performed as previously described (Su et al., 2019). Briefly, the mice were perfused with 0.9% NaCl and 4% PFA. The brain tissues were fixed in PFA at 4°C for 24 h and then dehydrated in 30% (w/v) sucrose in PFA at 4°C until complete dehydration. The brain slices were cut at 20-μm thickness on a cryostat. The probe for miR-144-3p was synthesized by TSINGKE (Wuhan, China), and FISH was performed according to the manufacturer’s instruction. All images were obtained with a confocal microscope (ZEISS, LSM 800).

### Administration of miR-144-3p Agomir and Antagomir

mmu-miR-144-3p agomir, antagomirs, and scrambled control were purchased from RiboBio (Guangzhou, China). Mice were anesthetized, and holes were made in the skull above the hippocampal CA3 (bregma: anterior/posterior −2.0 mm, medial/lateral ± 2.35 mm, and dorsal/ventral −2.35 mm). The concentration for miR-144-3p referenced previously published articles (Wang et al., 2018). About 1.5 μl of miR-144-3p antagomirs (50 μM) was stereotactically injected into the hippocampi of 11-months-old APP/PS1 mice every 2 weeks. The needle was left in the animal brain for 10 min, and it was then slowly withdrawn. Subsequently, the wound was sutured, and mice were allowed to recover.

### Enzyme-Linked Immunosorbent Assay (ELISA)

The NGF ELISA was performed by mouse an NGF ELISA kit (MBS702384, MyBioSource, Inc., United States) according to the manufacturer’s instructions. Briefly, 100 mg of tissue was homogenized in 1 ml of 1 × PBS. After two freeze–thaw cycles were performed to break the cell membranes, the homogenates were centrifuged at 5,000 g, 4°C for 5 min. The supernate was removed and assayed immediately. A 100 μl sample was added into a plate well and incubated for 2 h at 37°C. NGF was captured using NGF antibody-coated plates, followed by detection with biotinylated antibody. Samples were incubated with the secondary antibody and then with avidin horseradish peroxidase (HRP). Plates were developed using tetramethylbenzidine (TMB) as a substrate. After the reaction stopped, optical density was read at 450 nm. Raw data were converted to nanograms per gram of wet tissue by comparison to a standard curve of synthetic A.

### ChAT Activity Analysis

ChAT activity was determined by using a ChAT assay kit (Jiancheng Bioengineering Institute, Nanjing, China), following the manufacturer’s protocol. The tissue was removed from mice and homogenated in saline according to a weight–volume ratio of 5% (g/ml). The mixed solution was prepared at 37°C for 5 min according to the instructions, and a 25 μl of the sample was then added into the mixed solution at 37°C for 20 min. Subsequently, those samples were boiled at 100°C for 2 min to terminate the reaction, and 425 μl of double-distilled water was added. Then, samples were centrifuged at 4,000 rpm for 10 min, and 10 μl of solution 7 was added into 500 μl of supernatant for 15 min. Finally, optical density was measured at 324 nm. The ChAT activity (U/g tissue) was calculated according to the manufacturer’s instructions.

### Long-Term Potentiation (LTP) Recording

Mice were sacrificed, and the brains were quickly immersed in ice-cold artificial cerebrospinal fluid (ACSF) (in mM: 3.0 KCl, 2.5 CaCl_2_, 125 NaCl, 1.25 KH_2_PO_4_, 26 NaHCO_3_, 1.2 MgSO_4_, and 10 glucose), which was saturated with 95% oxygen and 5% carbon dioxide. Coronal brain slices of 300 μm were prepared with a vibratome (VT1000S, Leica, Germany). Brain sections were recovered in the oxygenated ACSF at 32°C for 30 min and then at room temperature for 1 h constantly immersed in ACSF. Then, brain sections were recorded by a planar multielectrode recording setup (MED64, Alpha Med Sciences, Japan). An electrophysiological recording method and statistical analysis were performed as previously reported (Wang et al., 2018).

### Luciferase Activity Assay

The wild-type or mutant tPA 3′-untranslated region (UTR) plasmid was cloned and inserted into psiCHECK-2 within *Xho*I and *Not*I restriction sites located downstream of the *Renilla* luciferase gene. All primers for cloning are provided as follows: wild-type tPA 3′-UTR (forward primer: 5′-CAA AGAAAGCCCAGCTCCTTC-3′, reverse primer: 5′-TTG GAAAAGTGTGAAAAATACCTC-3′); mutant tPA 3′-UTR (forward primer: 5′-GTATGTAATATCTCTTAAATAATAAATT CAGAGGTATTTTTCACA-3′, reverse primer: 5′-TAAGAGA TATTACATACAAAGTTATAGTAACAAAGTAAAAACTAAAA TAG-3′). Site-directed mutation of tPA 3′-UTR was performed by using Mut Express II Fast Mutagenesis Kit V2 (Vazyme, Nanjing, China). These plasmids were cotransfected into N2a cells with the miR-144-3p or the scramble agomir at a final concentration of 100 nM. After 48 h, cells were harvested, and lysates were used for firefly and *Renilla* luciferase activities using the dual-luciferase reporter assay kit (Promega) according to the manufacturer’s instruction. The normalized values (*Renilla*/firefly activity) were used for analysis. Experiments were performed in triplicate.

### Statistical Analysis

All data were shown as the mean ± SEM and analyzed using GraphPad Prism software (version 8). A two-tailed unpaired Student’s *t*-test was used to assess the variance between two groups, and the difference among multiple groups was analyzed by one-way ANOVA adjusted with Tukey’s multiple comparisons. Value with *p* < 0.05 are considered statistically significant. Both ^∗∗^*p* < 0.01 and ^∗∗∗^*p* < 0.001 represent extremely significant difference. All the statistical analysis data are supplied in Supplementary Table 2.

## Results

### Degeneration of the Cholinergic System Was Accompanied by Synaptic and Memory Impairments in APP/PS1 Mice

**FIGURE 1:**
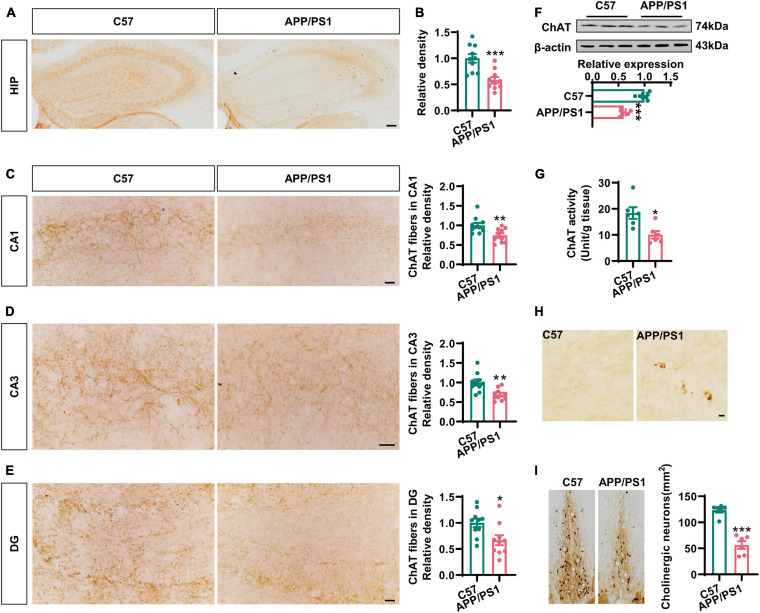
The cholinergic dysfunction was impaired in the hippocampi of AD mice. **(A)** Representative immunohistochemical images in the hippocampi stained with anti-ChAT antibody from APP/PS1 mice and age-matched C57 mice. Bar = 100 μm. **(B)** The relative density of ChAT fibers was analyzed by ImageJ. Unpaired Student’s *t*-test, *n* = 10 from four to five mice for each group, ***p < 0.001. **(C–E)** Representative immunohistochemical images in CA1, CA3, and DG stained with anti-ChAT antibody; the relative density of ChAT fibers was analyzed in CA1 **(C)**, CA3 **(D)**, and DG **(E)**. Bar = 20 μm. Unpaired Student’s *t*-test, *n* = 10 from six mice for each group, **p < 0.01 and *p < 0.05. **(F)** The protein level of ChAT in the hippocampi was measured by western blotting, and the quantitative analysis was performed in the lower panel. Unpaired Student’s *t*-test, *n* = 6 mice for each group, ***p < 0.001. **(G)** The ChAT enzyme activity in the hippocampi was measured by an ELISA kit. Unpaired Student’s *t*-test, *n* = 6 mice for each group, *p < 0.05. **(H)** Representative immunohistochemical images of dystrophic neurites in the hippocampi stained with anti-ChAT antibody from APP/PS1 mice and age-matched C57 mice. Bar = 10 μm. **(I)** Representative immunohistochemical images of ChAT-positive neurons in the basal nucleus of Meynert stained with anti-ChAT antibody. The quantitative analysis was performed and shown in the right panel. Bar = 50 μm. Unpaired Student’s *t*-test, *n* = 6 mice for each group, ***p < 0.001.

To investigate cholinergic dysfunction in a mouse model of AD, we first performed immunohistochemistry with an anti-ChAT antibody to identify cholinergic fibers in the hippocampi and the medial prefrontal cortex (mPFC), the two major regions of the brain that were projected from BFCNs (Ballinger et al., 2016). We found that the intensity of ChAT immunostaining was dramatically reduced in the hippocampi of 12-months-old AD mice when compared to that in the age-matched C57 controls (Figures 1A,B), thus indicating the loss of cholinergic fibers in AD. Specifically, the densities of cholinergic fibers had decreased to 74, 71, and 63% in the CA1, CA3, and DG areas of AD mice, respectively, when compared with wild-type controls (Figures 1C–E). In addition, the expression of ChAT protein and the levels of ChAT enzyme activity in the hippocampi were also significantly reduced in AD mice (Figures 1F,G). Similar results were also found in the mPFC region (Supplementary Figures 1A–C). Importantly, we observed some dystrophic neurites in the hippocampi and the mPFC of AD mice but not in control mice (Figure 1H and Supplementary Figure 1D). As expected, the loss of ChAT-positive neurons was obvious in the basal nucleus of Meynert (Figure 1I), which was consistent with previous report (Foidl et al., 2016). Collectively, these data strongly suggested that the degeneration of the cholinergic system occurs in the forebrain of AD mice.

**FIGURE 2:**
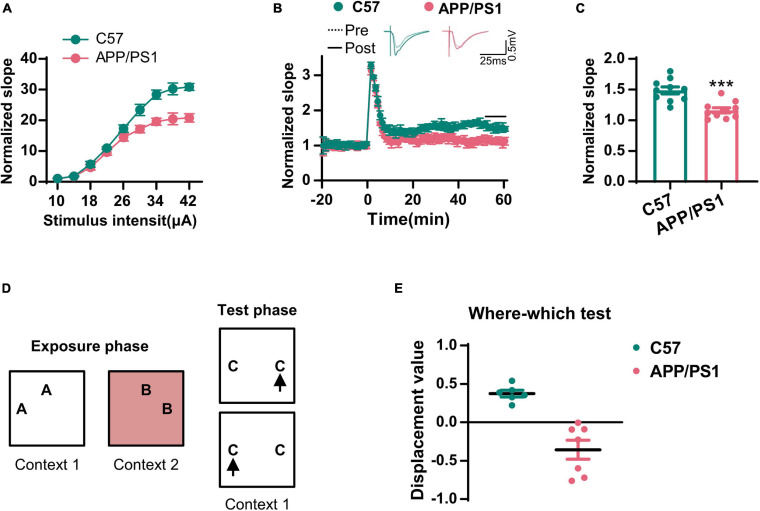
The synaptic transmission and context–place memory were derogated in AD mice. **(A)** The input–output curve of the CA3–CA1 LTP was shown. **(B)** The electrophysiological recording to detect the fEPSP slope was used to evaluate the CA3–CA1 LTP. The representative fEPSP traces were shown in the upper panel, and the relative fEPSP slopes were shown in the lower panel. **(C)** The fEPSP slope of the last 5 min was statistically analyzed. Unpaired Student’s *t*-test; *n* = 10 slices from three mice for each group, ***p < 0.001. **(D)** A schematic diagram of the where–which test was used to examine the context–place memory. **(E)** The displacement value in the where–which test was evaluated. Unpaired Student’s *t*-test, *n* = 6–7 mice for each group, ***p < 0.001.

The cholinergic system plays an important role in hippocampal synaptic plasticity and memory (Haam and Yakel, 2017). Next, we examined LTP in the CA3–CA1 synapses. We found that the input–output curve of the CA3–CA1 circuit was lower in the AD mice when compared to that in wild-type mice (Figure 2A). Following HFS stimulation, the normalized fEPSP was also reduced in the AD mice (Figures 2B,C). We also evaluated context–place memory by applying the where–which task (Figure 2D); this is a test that is known to rely on normal cholinergic functions (Easton et al., 2011). We found that wild-type mice exhibited exploration scores that were significantly greater than chance, while the AD mice did not (Figure 2E). These data suggested that synaptic plasticity and memory that were dependent on the cholinergic system were impaired in AD mice.

### Disturbance of the proNGF/NGF Ratio in AD Mice Was Caused by the Reduction of tPA and Plasmin

**FIGURE 3:**
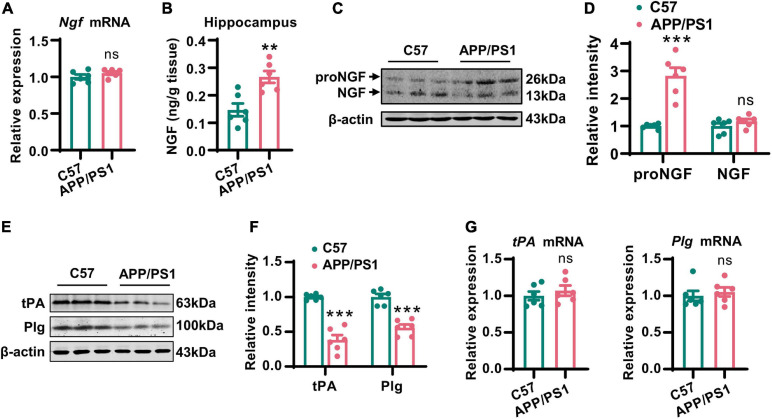
Disturbance of the proNGF/NGF ratio in AD mice was caused by the reduction of tPA and plasmin. **(A)** The NGF mRNA in hippocampi of 12-months-old APP/PS1 mice and C57 mice was examined by Q-PCR. Unpaired two-tailed Student’s *t*-test, *n* = 6 mice for each group; ns, no significance. **(B)** The total NGF in hippocampi of 12-months-old APP/PS1 mice and C57 mice was measured by an ELISA kit. Unpaired two-tailed Student’s *t*-test, *n* = 6 mice for each group, **p < 0.01. **(C,D)** The protein level of mature NGF in the hippocampi of 12-months-old APP/PS1 mice and C57 mice was detected by western blotting. The representative images were shown in **(C)**, and quantitative analysis was shown in **(D)**. Multiple *t*-test adjusted with the Holm-Šidák method, *n* = 6 mice for each group, ***p < 0.001. **(E,F)** The protein levels of tPA and plasminogen (Plg) were examined in the hippocampi of 12-months-old APP/PS1 mice and C57 mice. The representative images were shown in **(E)**, and quantitative analysis was shown in **(F)**. Multiple *t*-test adjusted with the Holm–Šidák method, *n* = 6 for each group, ***p < 0.001. **(G)** The mRNA levels of tPA and Plg in the hippocampi of 12-months-old APP/PS1 mice and C57 mice were examined by Q-PCR. Unpaired two-tailed Student’s *t*-test, *n* = 6 mice for each group; ns, no significance.

It has been established that mature NGF plays an important role in maintaining the normal function of the cholinergic system (Cuello et al., 2019). Therefore, we first examined the expression of NGF in the hippocampi by Q-PCR, ELISA, and western blotting. In line with previous studies (Fahnestock et al., 1996), the levels of mRNA encoding NGF did not change although the total level of NGF increased dramatically in AD mice (Figures 3A,B). This raised the question as to whether NGF deregulation is involved in the cholinergic degeneration of AD mice. We noted that the levels of proNGF, but not mature NGF, were increased in AD mice (Figures 3C,D), thus suggesting the disturbance of the proNGF/NGF ratio, an occurrence that has been validated in the brain of AD patients (Peng et al., 2004). Next, we investigated how the proNGF/NGF imbalance was induced. Considering these data, we predicted that an impairment of NGF maturation might be involved. As tPA and plasmin are the two most critical enzymes required for the maturation of NGF (Bruno and Cuello, 2006), we then examined the mRNA and protein levels of these two molecules. We found that the protein levels of tPA and plasmin were significantly lower in the hippocampi of AD mice than in the wild-type mice (Figures 3E,F); however, the levels of mRNA encoding tPA and plasmin remained unchanged (Figure 3G). Thus, the loss of tPA and plasmin might be crucial for disturbance in the proNGF/NGF ratio.

### Increased Expression of miR-144-3p Led to the Translational Repression of tPA

**FIGURE 4:**
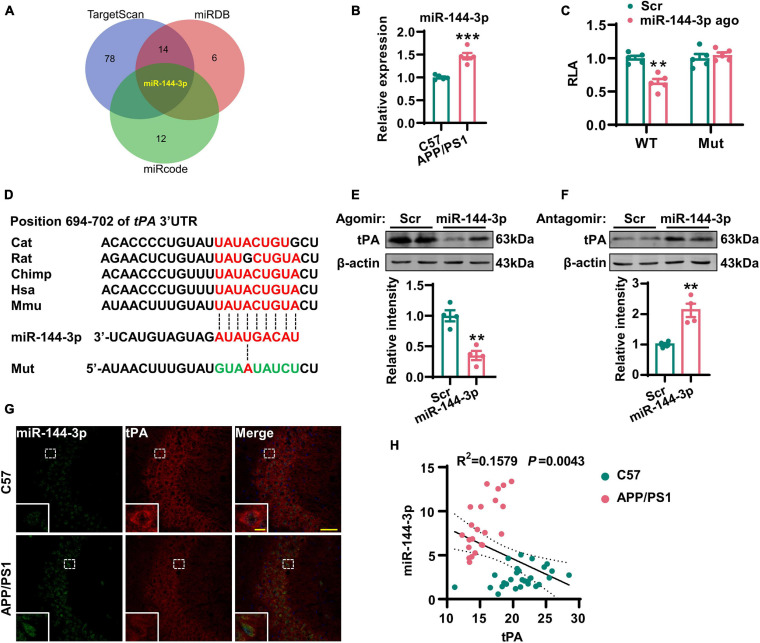
Increased expression of miR-144-3p led to the translational repression of tPA. **(A)** The miRNAs potentially regulating the tPA translation were shown in a Venn diagram by three different online tools as indicated. **(B)** The expression profiles of this candidate miRNA in the hippocampi of 12-months-old APP/PS1 mice and C57 mice were evaluated by Q-PCR. Unpaired Student’s *t*-test was used, *n* = 5 mice for each group, ***p < 0.001. **(C)** The wild-type (WT) or mutant (Mut) 3′-UTR of tPA in psiCHECK-2 vector was co-transfected into N2a cells with miR-144-3p agomir or scrambled control (Scr). The luciferase activity was determined at 48 h after the transfection. Multiple *t*-test adjusted with the Holm–Šidák method, *n* = 5 for each group, **p < 0.01. **(D)** Diagram to display the conserved binding site in tPA 3′-UTR to the miR-144-3p. The mutant sequence in 3′-UTR of tPA for luciferase analysis was provided at the bottom. **(E)** N2a cells were transfected with miR-144-3p agomir or Scr. The cell lysates were collected, and the protein levels of tPA were then detected 48 h later by western blotting. The quantitative analysis was shown in the lower panel. Unpaired Student’s *t*-test was used, *n* = 4 for each group, **p < 0.01. **(F)** N2a cells were transfected with miR-144-3p antagomir (miR-144 anta) or Scr. The protein levels of tPA were detected 48 h later by western blotting. The quantitative analysis was shown in the lower panel. Unpaired Student’s *t*-test, *n* = 4 for each group, **p < 0.01. **(G)** The staining of fluorescence *in situ* by using the probe of miR-144-3p (green) and immunofluorescence of anti-tPA (red) antibody was performed in the hippocampi of APP/PS1 mice and C57 mice at 12 months. Bar = 50 μm. The representative image of an amplified neuron was shown in the southwest corner. Bar = 5 μm. **(H)** The correlation analysis between the intensities of miR-144-3p and tPA in **(G)**. *n* = 22 or 28 from the APP/PS1 mice or C57 mice.

Considering that the loss of tPA was not due to the suppression of transcription, we next investigated whether posttranscriptional regulation might be involved. It is known that miRNAs are able to control gene expression at the posttranscriptional level by hybridizing to target mRNAs and thereby regulating their translation or stability (Jonas and Izaurralde, 2015). Previous researchers have reported alterations in a range of miRNAs in the AD brain (Wang et al., 2019). Therefore, we attempted to investigate whether any of these miRNAs may play an important role in the posttranscriptional repression of tPA. By using three online prediction tools (TargetScan, miRDB, and miRcode), we found that miR-144-3p may represent a key regulatory miRNA for tPA and plasmin (Figure 4A, Supplementary Figure 2, and Supplementary Table 1). Next, we investigated the levels of miR-144-3p in the hippocampi and found that levels of this miRNA were increased in AD mice (Figure 4B). By using a luciferase reporter system, we identified that miR-144-3p is able to bind with the wild-type 3′-UTR of tPA. However, when the seed-region binding site in the 3′-UTR of tPA was mutated, miR-144-3p cannot bind with it (Figure 4C). Furthermore, the binding site for miR-144-3p in the 3′-UTR of tPA is conserved in mammalian species (Figure 4D). In N2a cells, the application of the agomir or antagomir of miR-144-3p was able to downregulate or upregulate the protein levels but not the mRNA levels of tPA (Figures 4E,F and Supplementary Figure 3). In the hippocampi of AD mice, we observed a negative correlation between the levels of miR-144-3p and those of tPA (Figures 4G,H). Moreover, in the mPFC of AD mice, the levels of miR-144-3p were also increased and negatively correlated with the levels of tPA (Supplementary Figures 4A–C). Collectively, these data suggested that miR-144-3p regulates the levels of tPA in a direct manner.

### The Administration of miR-144-3p Induced Cholinergic Degeneration and Synaptic/Memory Impairments

**FIGURE 5:**
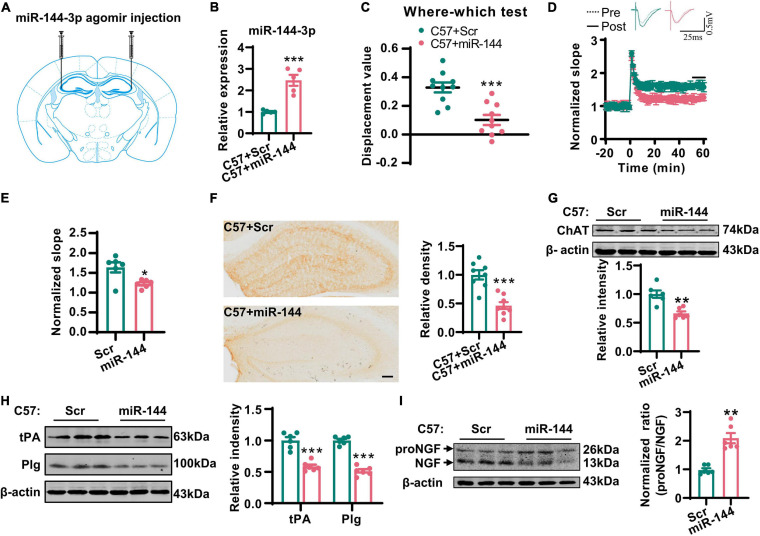
Overexpression of miR-144-3p induced cholinergic degeneration and synaptic/memory impairments. **(A)** The diagram for the stereotactic injection experiment was shown. **(B)** The expression of miR-144-3p was evaluated in 3-months-old C57 mice after injection of miR-144-3p agomir 2 weeks later. Unpaired Student’s *t*-test, *n* = 5 mice for each group, **p < 0.01. **(C)** The where–which test was used to assess the context memory after administration of miR-144-3p agomir. Unpaired Student’s *t*-test, *n* = 10 mice for each group, ***p < 0.001. **(D)** The electrophysiological recording to detect the fEPSP slope was used to evaluate CA3–CA1 LTP after administration of miR-144-3p agomir. The representative fEPSP traces were shown in the upper panel, and the relative fEPSP slopes were shown in the lower panel. *n* = 4–6 slices from three mice for each group. **(E)** The fEPSP slope of the last 5 min was statistically analyzed. Unpaired Student’s *t*-test, *n* = 6 from three mice for each group, *p < 0.05. **(F)** Representative immunohistochemical images in the hippocampi in C57 mice with miR-144-3p agomir treatment. And the quantitative analysis was shown in the right panel. Bar = 100 μm. Unpaired Student’s *t*-test, *n* = 8 from four mice for each group, ***p < 0.001. **(G)** Hippocampal tissue lysates from the mice above were collected, and the protein level of ChAT was detected by western blotting. Unpaired Student’s *t*-test, *n* = 6 mice for each group, **p < 0.01. **(H)** The protein levels of tPA and Plg were also detected in those mice by western blotting, and the quantitative analysis was shown in the right panel. Unpaired Student’s *t*-test, *n* = 6 mice for each group, ***p < 0.001. **(I)** The protein level of NGF was examined by western blotting in the left panel, and the normalized proNGF/NGF ratio was shown in the right panel. Unpaired Student’s *t*-test, *n* = 6 mice for each group, **p < 0.01.

Next, we investigated whether the artificial upregulation of miR-144-3p could induce an imbalance of the proNGF/NGF ratio in the hippocampi and then lead to cholinergic degeneration and synaptic/memory impairment. To this end, we injected the agomir of miR-144-3p or the scrambled control into the hippocampi of wild-type mice at 3 months old (Figures 5A,B). Two weeks later, we subjected the mice to the where–which task. We found that the discrimination index was reduced in the mice treated with miR-144-3p (Figure 5C). Electrophysiological recordings suggested that the LTP of the CA3–CA1 circuit was also reduced in the mice treated with miR-144-3p (Figures 5D,E). We also noted that the upregulation of miR-144-3p induced a lower intensity of ChAT immunostaining in the hippocampi (Figure 5F) and decreased the expression of ChAT protein (Figure 5G). Furthermore, the levels of tPA and plasmin were decreased in the hippocampi of mice treated with miR-144-3p (Figure 5H). Importantly, the proNGF/NGF ratio was increased in the mice treated with miR-144-3p (Figure 5I). These data suggested that the artificial administration of miR-144-3p could induce cholinergic degeneration and synaptic/memory impairments by elevating the proNGF/NGF ratio.

### The Inhibition of miR-144-3p Rescued Cholinergic Degeneration and Synaptic/Memory Impairments in a Mouse Model of AD

**FIGURE 6:**
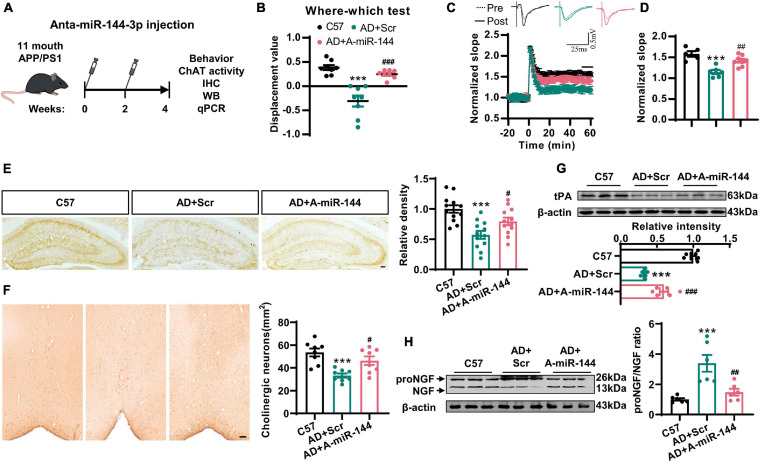
The inhibition of miR-144-3p rescued cholinergic degeneration and synaptic/memory impairments in APP/PS1 mice. **(A)** The flow diagram of administration of miR-144-3p antagomir. The miR-144-3p antagomir was injected into the hippocampal CA3 of 11-months-old APP/PS1 mice that were used for subsequent experiments. **(B)** The where–which test was used to assess the context memory in C57, APP/PS1 + Scr (AD + Scr) and APP/PS1 + A-miR-144 (AD + A-miR-144) groups. Ordinary one-way ANOVA with Tukey’s multiple comparisons, *n* = 8 mice for each group, ***p < 0.001 for AD + Scr vs. C57 and ^###^*p* < 0.001 for AD + A-miR-144 vs. AD + Scr. **(C)** The electrophysiological recording to detect the fEPSP slope was used to evaluate the CA3–CA1 LTP of those three groups. The representative fEPSP traces were shown in the upper panel, and the relative fEPSP slopes were shown in the lower panel. *n* = 5–8 slices from three mice for each group. **(D)** The fEPSP slope of the last 5 min was statistically analyzed. *n* = 5–8 from three mice for each group, ***p < 0.001 for AD + Scr vs. C57 and ^##^*p* < 0.01 for AD + A-miR-144 vs. AD + Scr. **(E)** Representative immunohistochemical images in the hippocampi from mice in those three groups were shown in the left panel, and the quantitative analysis was shown in the right panel. Bar = 100 μm. Ordinary one-way ANOVA with Tukey’s multiple comparisons, *n* = 12 from four mice for each group, ***p < 0.001 for AD + Scr vs. C57 and ^#^*p* < 0.05 for AD + A-miR-144 vs. AD + Scr. **(F)** Representative immunohistochemical images of ChAT-positive neurons in the basal nucleus of Meynert were shown. Bar = 100 μm. The quantitative analysis was indicated in the right panel. Ordinary one-way ANOVA with Tukey’s multiple comparisons, *n* = 8 from four mice for each group, ***p < 0.001 for AD + Scr vs. C57 and ^#^*p* < 0.05 for AD + A-miR-144 vs. AD + Scr. **(G)** The protein level of tPA was detected in those mice by western blotting, and the quantitative analysis was shown. Ordinary one-way ANOVA with Tukey’s multiple comparisons, *n* = 6 mice for each group, ***p < 0.001 for AD + Scr vs. C57 and ^###^*p* < 0.001 for AD + A-miR-144 vs. AD + Scr. **(H)** The protein level of tPA was detected in the left panel, and the quantitative analysis was shown in the right panel. Ordinary one-way ANOVA with Tukey’s multiple comparisons, *n* = 6 mice for each group, ***p < 0.001 for AD + Scr vs. C57 and ^##^*p* < 0.01 for AD + A-miR-144 vs. AD + Scr.

Finally, we investigated whether the inhibition of miR-144-3p could rescue cholinergic degeneration and synaptic/memory impairments in a mouse model of AD. We injected the antagomir of miR-144-3p into the hippocampi of 11-months-old APP/PS1 mice every 2 weeks. One month later, the high levels of miR-144-3p in APP/PS1 mice were significantly reduced by the inhibition of miR-144-3p (Supplementary Figure 5). Subsequently, we found that the inhibition of miR-144-3p (Figure 6A) significantly restored the discrimination index derived from the where–which task in the APP/PS1 mice (Figure 6B). We also observed that the LTP in the CA3–CA1 circuit was also rescued by the inhibition of miR-144-3p (Figures 6C,D). ChAT immunoreactivity in the hippocampi, along with neuronal loss in the basal nucleus of Meynert, was also restored (Figures 6E,F). Furthermore, the loss of tPA was restored, and the proNGF/NGF ratio was suppressed in mice treated with the miR-144-3p antagomir (Figures 6G,H). Thus, the inhibition of miR-144-3p was able to rescue cholinergic degeneration and synaptic/memory impairments in the mouse model of AD.

## Discussion

In this study, we demonstrated that cholinergic degeneration is accompanied by synaptic disorders in the hippocampi and memory impairments in AD mice. One of the most prominent clinical symptoms of AD patients is the progressive decline in spatial memory. More specifically, these patients persistently forget where they are or how they arrived at particular locations (Alzheimer’s Disease Facts and Figures, 2020). A previous clinical study recruited 31 patients with AD and 35 healthy aged-matched controls; a context memory task revealed that the AD patients exhibited difficulties in remembering information related to “who,” “where,” and “when” (El Haj and Antoine, 2018). Previous studies suggested that compromised function in the hippocampi and prefrontal cortex may contribute to contextual memory errors in patients with AD (Mitchell et al., 2006). Here, we demonstrated cholinergic degeneration, such as the loss of cholinergic fibers, the formation of dystrophic neurites, and the reduction of ChAT activity, in the hippocampi and mPFC of AD mice with contextual memory impairments. It is known that the hippocampi and mPFC are the two key brain regions that receive cholinergic projections from the BFCN. Previous research showed that the impairment of cholinergic neurons in the medial septum of rats led to a worse performance in where–which memory (Easton et al., 2011). In addition, acetylcholine in the hippocampi is able to encode novel contexts into a higher priority by controlling sensory inputs from proactive inhibition (Maurer and Williams, 2017). Therefore, cholinergic degenerations in the hippocampi might play an important role in the contextual memory impairments in AD mice. By reversing the cholinergic degeneration induced by the suppression of miR-144-3p, we found that we were able to rescue the contextual memory impairment.

The deregulation of miRNAs has been well validated in AD (Liu et al., 2017; Wang et al., 2018; Tang et al., 2019; Xie et al., 2019; Hou et al., 2020). In the present study, we found that miR-144-3p was upregulated in the hippocampi and mPFC of mice with AD. The injection of the agomir of miR-144-3p into the hippocampi led to cholinergic degeneration, hippocampal synaptic disorders, an imbalance of the proNGF/NGF ratio, and contextual memory deficits, as seen in patients with AD. In line with our current data, previous work showed that miR-144-3p can be stimulated by Aβ and then suppress the expression of A disintegrin and metalloprotease 10 (ADAM10) (Cheng et al., 2013). The reduction of ADAM10 promoted the generation of Aβ and formed a vicious cycle. And this report also indicated that transcription factor AP-1 promoted the expression of miR-144 through the AP-1 binding sites located upstream of the miR-144 precursor. Previous research has also reported increased expression levels of miR-144-3p in the plasma of patients with traumatic brain injury (TBI) and rat models of TBI. The inhibition of miR-144-3p exerts protective effects on the rat model of TBI, including the reduction of lesion volume and brain edema, and also recovered cognitive deficits (Sun et al., 2017). miR-144-3p has also been implicated in many other neurological disorders. For example, miR-144-3p was shown to be an extinction-specific regulated miRNA due to the fact that the expression of miR-144-3p was increased in the amygdala of both extinction-intact BL6 mice and extinction-rescued 129S1/SvlmJ mice (Murphy et al., 2017). Researchers have also discovered that the plasma levels of miR-144-3p were reduced in patients suffering from depression. Interestingly, after 8 weeks of psychotherapy, the expression levels of miR-144-5p were restored to normal, as seen in healthy controls (Wang et al., 2015). A genome-wide study suggested that miR-144-3p plays a crucial role in brain aging and the pathogenesis of spinocerebellar ataxia (Persengiev et al., 2011). We also identified that the increased levels of miR-144-3p inhibit the translation of tPA and subsequently lead to an imbalance of the proNGF/NGF ratio in AD mice. Indeed, a number of validated targets for miR-144-3p have been associated with signals related to synaptic plasticity, including the PI3K/AKT (Jiang et al., 2015), MAPK/ERK (Li et al., 2014), and Notch signaling pathways (Sureban et al., 2011). Interestingly, NGF has been linked with the PI3K/AKT (Sang et al., 2018), MAPK/ERK (Karmarkar et al., 2011), and Notch signaling pathways (Salama-Cohen et al., 2006) and can independently stimulate neuronal projections, neuroprotection, and synaptic inputs. Thus, maintaining the appropriate expression levels of miR-144-3p might be very important with regard to NGF and related signaling pathways.

NGF is a neurotrophin that is highly conserved across vertebrates (Ullrich et al., 1983). The maturation of NGF results from the cleavage of the NGF precursor and forms a biologically active dimer (Iulita and Cuello, 2014). Its mRNA has been shown to exist in the neocortex and hippocampi, while relatively high NGF protein levels have been detected in the cell body of neurons in the basal forebrain and septum (Korsching et al., 1985). Cholinergic neurons in the basal forebrain require NGF in order to maintain functional capability; NGF can be transported in a retrograde manner from the fibers to innervate cholinergic neurons (Hamburger and Levi-Montalcini, 1949). Research has shown that the expression of NGF was positively correlated with the levels of ChAT and AChE during postnatal development (Isaev et al., 2017). Furthermore, cholinergic neurons were shown to be impaired in NGF-knockout mice (NGF^+ /−^); this was accompanied by memory and learning deficits (Chen et al., 1997). Furthermore, the number of cholinergic neurons was dramatically reduced in AD (Yan et al., 2018), while the application of NGF in AD mice prevented cholinergic deficit, β-amyloid accumulation, and memory loss (Eyjolfsdottir et al., 2016; Yan et al., 2018). Collectively, these reports were consistent with our findings in that the indirect restoration of NGF by the injection of an antagomir of miR-144-3p could rescue cholinergic metabolic dysfunction and contextual memory in AD mice.

**FIGURE 7:**
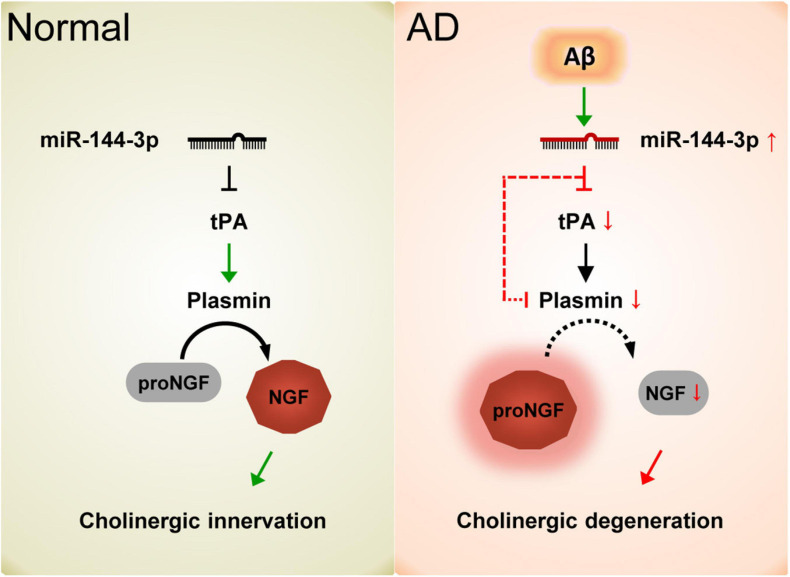
Schematic diagram of the current study. In normal conditions, the proper levels of tPA and plasmin play an important role in the maturation of NGF and maintain the function of the cholinergic system. In AD, the elevation of miR-144-3p leads to the downregulation of tPA and plasmin, which in turn inhibits the maturation of NGF and impairs the plasticity of cholinergic neurons.

Collectively, our data demonstrated that the elevation of miR-144-3p may play an important role in the imbalance of the proNGF/NGF ratio and that this event subsequently leads to cholinergic degeneration and synaptic/memory impairments in AD (Figure 7).

## Data Availability Statement

The raw data supporting the conclusions of this article are included in the article/Supplementary Material, without undue reservation.

## Ethics Statement

The animal study was reviewed and approved by the Huazhong University of Science and Technology.

## Author Contributions

DL initiated and designed the study. L-QZ, DL, and CY supervised the study. L-TZ, JZ, LT, H-ZH, YZ, and Z-QL performed the molecular biological experiments and animal experiments. L-TZ, JZ, and LT analyzed the data. DL and L-TZ wrote the manuscript. All authors contributed to the article and approved the submitted version.

## Conflict of Interest

The authors declare that the research was conducted in the absence of any commercial or financial relationships that could be construed as a potential conflict of interest.
