# Supplementary material for: Elevated Levels of miR-144-3p Induce Cholinergic Degeneration by Impairing the Maturation of NGF in Alzheimer’s Disease
Source: Front Cell Dev Biol. 2021 Apr 9;9:667412. doi: 10.3389/fcell.2021.667412 (PMC8063700; doi:10.3389/fcell.2021.667412)

Supplementary Fig 1

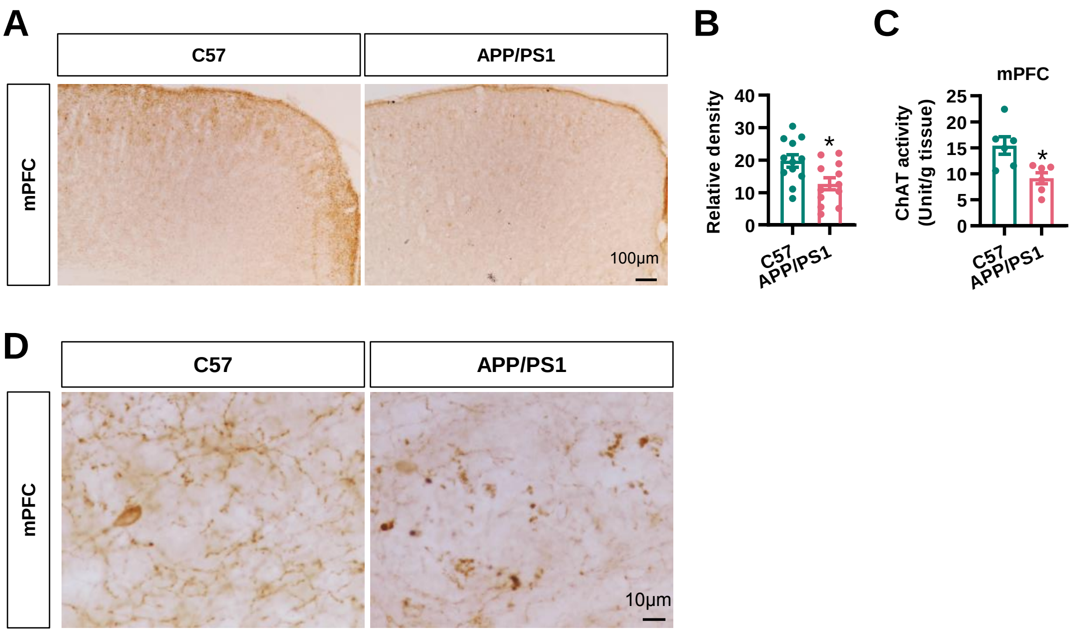

Supplementary Fig 2

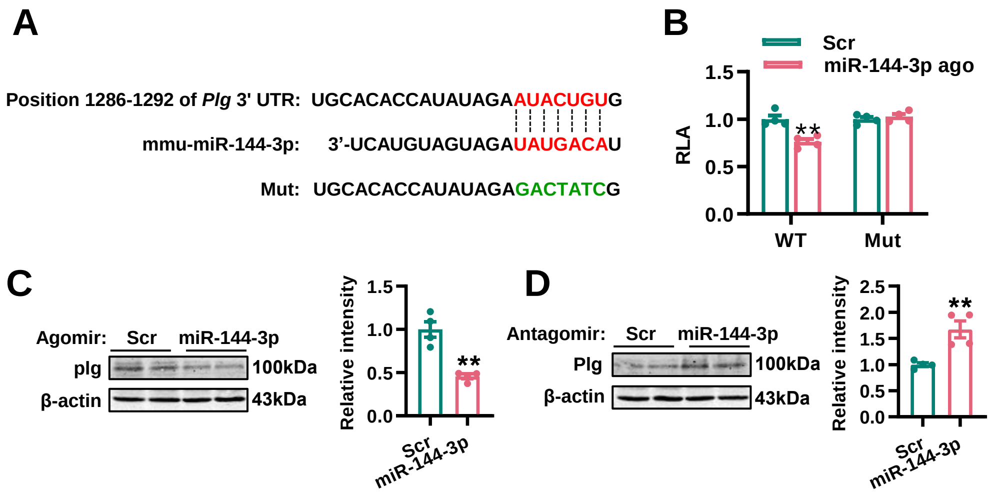

Supplementary Fig 3

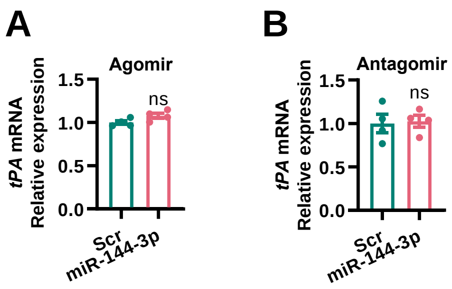

Supplementary Fig 4

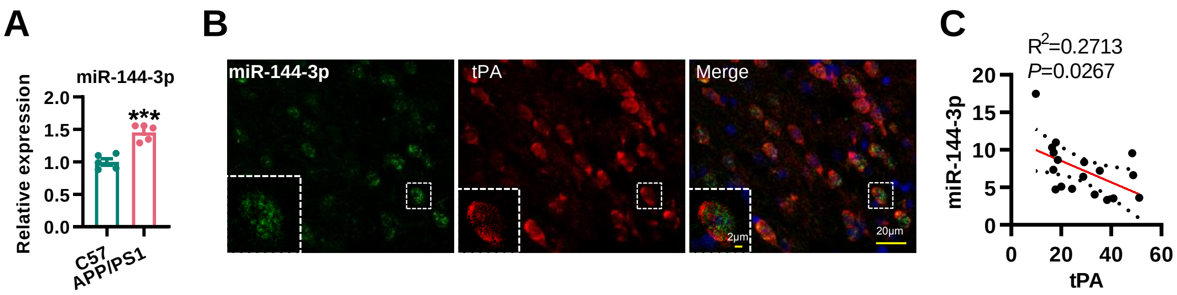

Supplementary Fig 5

A

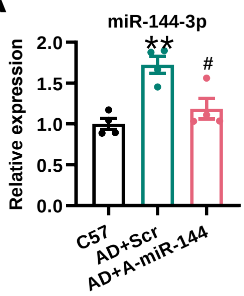

Supplement: Supplementary file 1 [file Data_Sheet_1.PDF]
