# Supplementary material for: Elevated Levels of miR-144-3p Induce Cholinergic Degeneration by Impairing the Maturation of NGF in Alzheimer’s Disease
Source: Front Cell Dev Biol. 2021 Apr 9;9:667412. doi: 10.3389/fcell.2021.667412 (PMC8063700; doi:10.3389/fcell.2021.667412)
Supplement: Supplementary file 3 [file Data_Sheet_3.docx]

**Supplementary Table2 Statistics.**

**Fig 1-6**

| **Fig** | **Statistic Method** | **Comparison** | **Number** | **Statistics** | **P Value** |
| --- | --- | --- | --- | --- | --- |
| **1B** | Unpaired student’s *t*-test | APP/PS1 *vs* C57 | 10,10 |  | 0.0006 |
| **1C** | Unpaired student’s *t*-test | APP/PS1 *vs* C57 | 10,10 |  | 0.0081 |
| **1D** | Unpaired student’s *t*-test | APP/PS1 *vs* C57 | 10,10 |  | 0.0025 |
| **1E** | Unpaired student’s *t*-test | APP/PS1 *vs* C57 | 10,10 |  | 0.0169 |
| **1F** | Unpaired student’s *t*-test | APP/PS1 *vs* C57 | 6,6 |  | <0.0001 |
| **1G** | Unpaired student’s *t*-test | APP/PS1 *vs* C57 | 6,6 |  | 0.0106 |
| **1I** | Unpaired student’s *t*-test | APP/PS1 *vs* C57 | 6,6 |  | <0.0001 |
| **2A** | Two-way ANOVA, Sidak post hoc | -  APP/PS1 *vs* C57 | 1,4 (Fn, Fd)  3,3 | F=33.90 | 0.0043 |
| **2C** | Unpaired student’s *t*-test | APP/PS1 *vs* C57 | 10,10 |  | 0.0002 |
| **2E** | Unpaired student’s *t*-test | APP/PS1 *vs* C57 | 6,7 |  | 0.0003 |
| **3A** | Unpaired student’s *t*-test | APP/PS1 *vs* C57 | 6,6 |  | 0.2051 |
| **3B** | Unpaired student’s *t*-test | APP/PS1 *vs* C57 | 6,6 |  | 0.0035 |
| **3D** | Multiple t test adjusted with Holm-Sidak | proNGF  NGF | 6,6 |  | <0.0001  0.2190 |
| **3F** | Multiple t test adjusted with Holm-Sidak | tPA  Plg | 6,6 |  | <0.0001  <0.0001 |
| **3G** | Unpaired student’s *t*-test | tPA  Plg | 6,6 |  | 0.4508  0.5767 |
| **4B** | Unpaired student’s *t*-test | APP/PS1 *vs* C57 | 5,5 |  | 0.0003 |
| **4C** | Multiple t test adjusted with Holm-Sidak | WT  Mut | 5,5 |  | <0.0001  0.5249 |
| **4E** | Unpaired student’s *t*-test | Scr *vs* miR-144-3p | 4,4 |  | 0.0015 |
| **4F** | Unpaired student’s *t*-test | Scr *vs* miR-144-3p | 4,4 |  | 0.0025 |
| **4H** | Linear regression |  | R^2^=0.1579 |  | 0.0043 |
| **5B** | Unpaired student’s *t*-test | C57+Scr *vs* C57+miR-144 | 5,5 |  | 0.0004 |
| **5C** | Unpaired student’s *t*-test | C57+Scr *vs* C57+miR-144 | 10,10 |  | 0.0002 |
| **5E** | Unpaired student’s *t*-test | C57+Scr *vs* C57+miR-144 | 6,6 |  | 0.0111 |
| **5F** | Unpaired student’s *t*-test | C57+Scr *vs* C57+miR-144 | 8,8 |  | 0.0001 |
| **5G** | Unpaired student’s *t*-test | C57+Scr *vs* C57+miR-144 | 6,6 |  | 0.0011 |
| **5H** | Multiple t test adjusted with Holm-Sidak | tPA  Plg | 6,6 |  | <0.0001  <0.0001 |
| **5I** | Unpaired student’s *t*-test | C57+Scr *vs* C57+miR-144 | 6,6 |  | 0.0002 |
| **6B** | One way ANOVA, Tukey's post hoc | -  AD+Scr *vs* C57  AD+A-miR-144 *vs* AD+Scr | 2,21 (Fn, Fd)  8,8  8,8 | F=24.60 | <0.0001  <0.0001  <0.0001 |
| **6D** | One way ANOVA, Tukey's post hoc | -  AD+Scr *vs* C57  AD+A-miR-144 *vs* AD+Scr | 2,16 (Fn, Fd)  6,5  8,6 | F=15.24 | 0.0002  0.0002  0.0047 |
| **6E** | One way ANOVA, Tukey's post hoc | -  AD+Scr *vs* C57  AD+A-miR-144 *vs* AD+Scr | 2,33 (Fn, Fd)  12,12  12,12 | F=11.26 | 0.0002  0.0001  0.0492 |
| **6F** | One way ANOVA, Tukey's post hoc | -  AD+Scr *vs* C57  AD+A-miR-144 *vs* AD+Scr | 2,21 (Fn, Fd)  8,8  8,8 | F=10.81 | 0.0006  0.0005  0.0196 |
| **6G** | One way ANOVA, Tukey's post hoc | -  AD+Scr *vs* C57  AD+A-miR-144 *vs* AD+Scr | 2,15 (Fn, Fd)  6,6  6,6 | F=69.94 | <0.0001  <0.0001  0.0008 |
| **6H** | One way ANOVA, Tukey's post hoc | -  AD+Scr *vs* C57  AD+A-miR-144 *vs* AD+Scr | 2,15 (Fn, Fd)  6,6  6,6 | F=10.81 | 0.0005  0.0006  0.0039 |

**Supplementary Fig 1-5**

| **Fig** | **Statistic Method** | **Target** | **Number** | **Statistics** | **P Value** |
| --- | --- | --- | --- | --- | --- |
| **1B** | Unpaired student’s *t*-test | APP/PS1 *vs* C57 | 12,12 |  | 0.0145 |
| **1C** | Unpaired student’s *t*-test | APP/PS1 *vs* C57 | 6,6 |  | 0.0119 |
| **2B** | Multiple t test adjusted with Holm-Sidak | WT  Mut | 4,4 |  | 0.0033  0.4999 |
| **2C** | Unpaired student’s *t*-test | Scr *vs* miR-144-3p | 4,4 |  | 0.0013 |
| **2D** | Unpaired student’s *t*-test | Scr *vs* miR-144-3p | 4,4 |  | 0.0064 |
| **3A** | Unpaired student’s *t*-test | Scr *vs* miR-144-3p | 4,4 |  | 0.0794 |
| **3B** | Unpaired student’s *t*-test | Scr *vs* miR-144-3p | 4,4 |  | 0.8502 |
| **4A** | Unpaired student’s *t*-test | APP/PS1 *vs* C57 | 5,5 |  | 0.0003 |
| **4B** | Linear regression |  | R^2^=0.2713 |  | 0.0267 |
| **5A** | One way ANOVA, Tukey's post hoc | -  AD+Scr *vs* C57  AD+A-miR-144 *vs* AD+Scr | 2,9 (Fn, Fd)  4,4  4,4 | F=13.40 | 0.0020  0.0020  0.0122 |
