# Supplementary material for: Elevated Levels of miR-144-3p Induce Cholinergic Degeneration by Impairing the Maturation of NGF in Alzheimer’s Disease
Source: Front Cell Dev Biol. 2021 Apr 9;9:667412. doi: 10.3389/fcell.2021.667412 (PMC8063700; doi:10.3389/fcell.2021.667412)
Supplement: Supplementary file 4 [file Data_Sheet_4.docx]

Supplementary Material

# Supplementary Figures


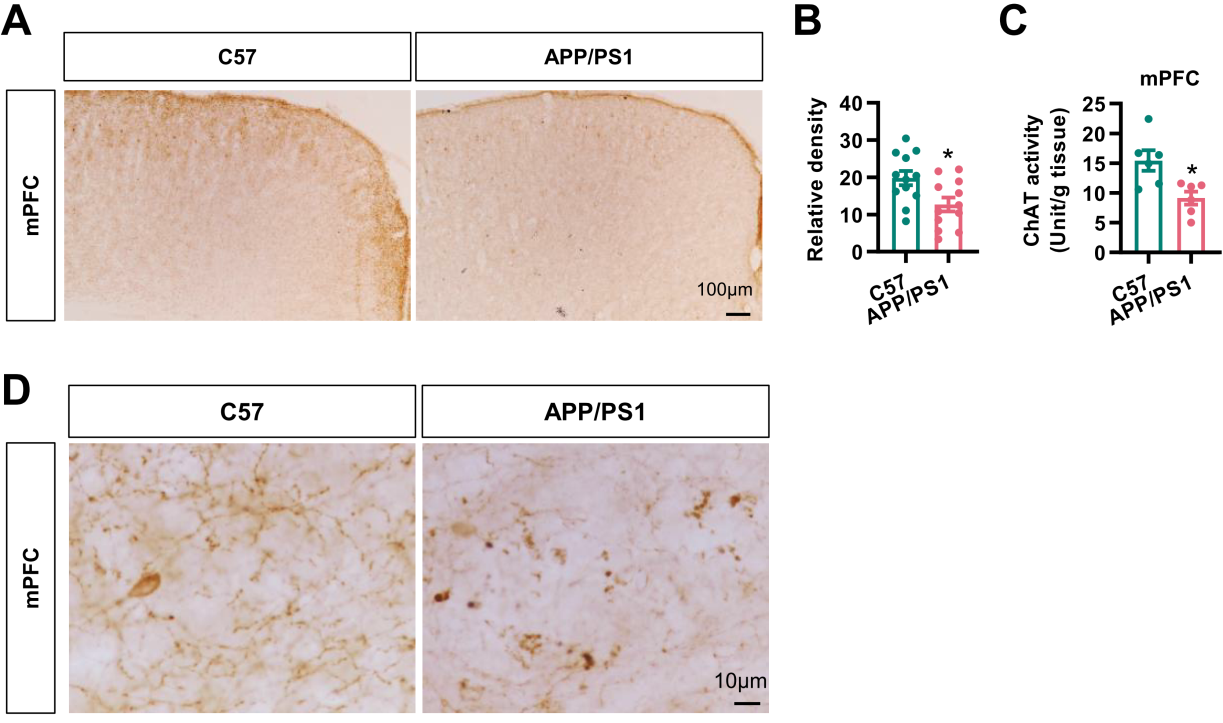


**Supplementary Figure 1 The cholinergic dysfunction was impaired in the prefrontal cortex of AD mice.**

(A, B) Representative immunohistochemical images in the prefrontal cortex (mPFC) stained with anti-ChAT antibody from APP/PS1 mice and age matched C57 mice. Bar = 100 μm. The relative density of ChAT fibers was analyzed by Image J (B). Unpaired student’s *t*-test, n= 12 from 4 mice for each group, **p* <0.05.

(C) The ChAT enzyme activity in the mPFC was measured by ELISA. Unpaired student’s *t*-test, n= 6 mice for each group, **p* <0.05.

(D) Representative immunohistochemical images of dystrophic neurites in the mPFC stained with anti-ChAT antibody. Bar = 10 μm.

**
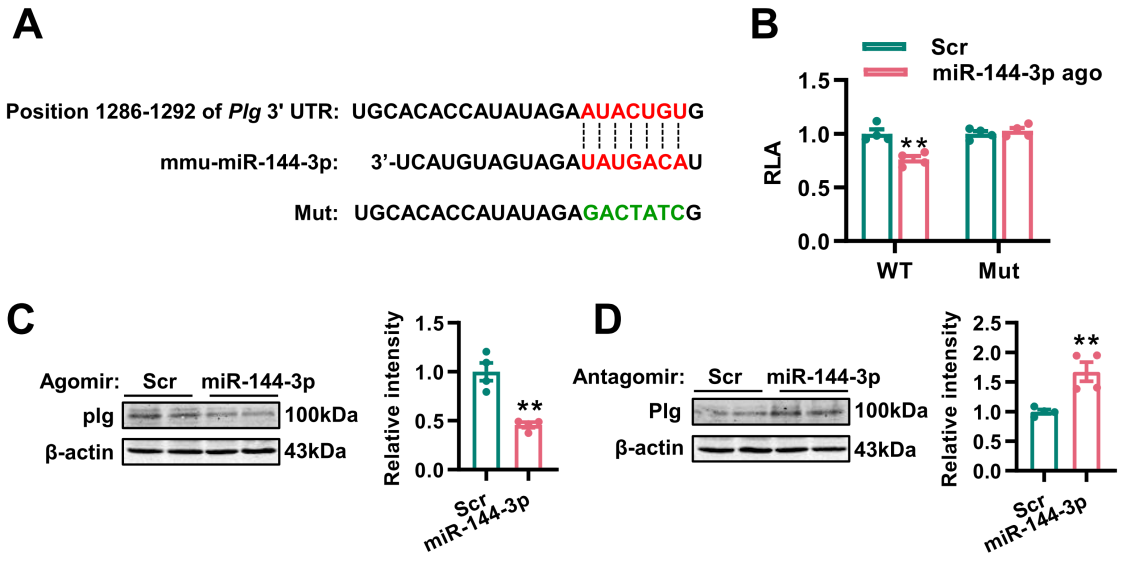
**

**Supplementary Figure 2 miR-144-3p also regulated its target gene, Plg, *in vitro***

(A) Diagram to display the binding site in *Plg* 3’UTR to the miR-144-3p. The mutant sequence in 3’UTR of *Plg* for luciferase analysis was provided at the bottom.

(B) The wild type (WT) or mutant (Mut) 3’UTR of *Plg* in psiCHECK-2 vector was co-transfected into N2a cells with miR-144-3p agomir or scrambled control (Scr). The luciferase activity was determined at 48h after the transfection. Multiple t test adjusted with Holm-Sidak method, n = 4 for each group, ***p* < 0.01.

(C) N2a cells were transfected with miR-144-3p agomir or scrambled control. The cell lysates were collected and the protein levels of Plg were then detected at 48 hours later by western blotting. The quantitative analysis was shown in the right panel. Unpaired student’s *t*-test was used, n = 4 for each group, ***p* < 0.01.

(D) N2a cells were transfected with miR-144-3p antagomir (miR-144 anta) or scrambled control (Scr). The protein levels of Plg were detected at 48 hours later by western blotting. The quantitative analysis was shown in right panel. Unpaired student’s *t*-test, n = 4 for each group, ***p* < 0.01.

**
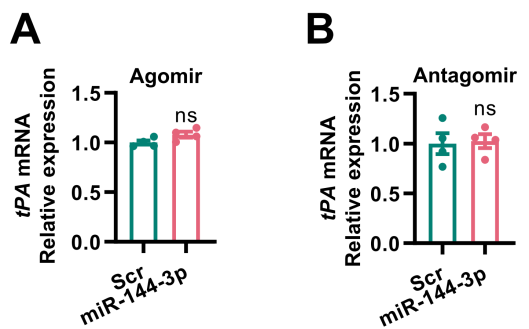
**

**Supplementary Figure 3 The expression of tPA mRNA was detected in N2a treated with miR-144-3p agomir or antagomir**

(A) N2a cells were transfected with miR-144-3p agomir or scrambled control. The cell lysates were collected and the expression of *tPA* mRNA was then detected at 48 hours later by Q-PCR. Unpaired student’s *t*-test was used, n = 4 for each group, ns, no significance.

(B) N2a cells were transfected with miR-144-3p antagomir or scrambled control. The cell lysates were collected and the expression of *tPA* mRNA was then detected at 48 hours later by Q-PCR. Unpaired student’s *t*-test was used, n = 4 for each group, ns, no significance.


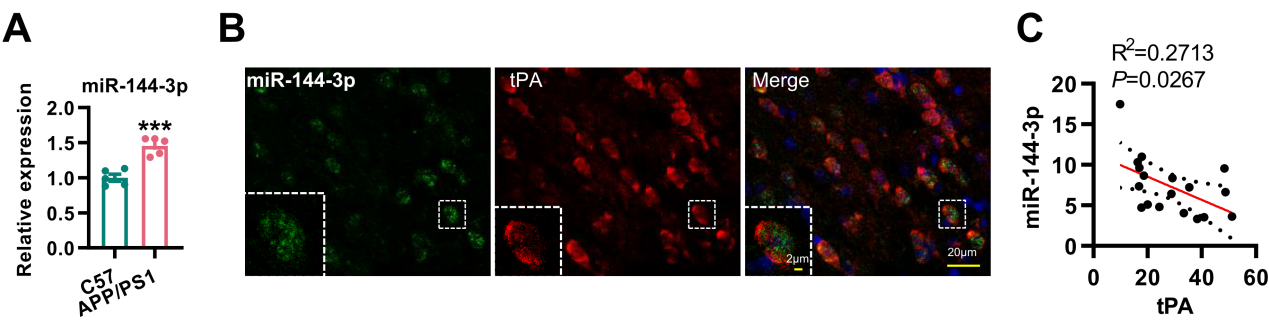


**Supplementary Figure 4 The levels of miR-144-3p were also increased and negatively correlated with the levels of tPA in the mPFC**

(A) The expression of miR-144-3p in mPFC of APP/PS1 mice and C57 mice at 12 months was evaluated by Q-PCR. Unpaired student’s *t*-test, n = 6 mice, ****p* < 0.001.

(B) Representative image of fluorescence in situ by using the probe of miR-144-3p (Green) and immunofluorescence of anti-tPA (Red) antibody in the mPFC of AD mice (Bar = 20 μm). An amplified neuron was shown in the southwest corner (Bar=2 μm).

(C) The correlation analysis was performed between the intensities of miR-144-3p and tPA in (B). n = 18 from 6 mice.

**
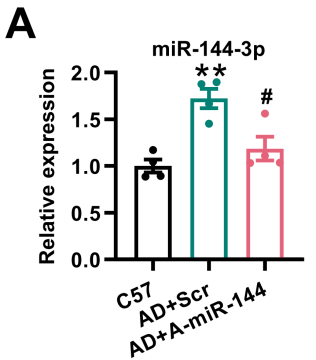
**

**Supplementary Figure 5 The expression levels of miRNA-144-3p was detected in the APP/PS1 mice treated with miR-144-3p antagomir**

(A) The expression levels of miR-144-3p was detected in C57 mice, APP/PS1+Scramble (AD+Scr), APP/PS1+miR-144 antagomir (AD+A-miR-144). Ordinary one-way ANOVA with Tukey's multiple comparisons, n = 4 mice for each group, ***p* < 0.01 for AD+Scr vs C57, #*p* < 0.05 for AD+A-miR-144 vs AD+Scr.
